# Health-Related Quality of Life Among Irregular Sub-Saharan Migrants in Northern Morocco

**DOI:** 10.7759/cureus.67457

**Published:** 2024-08-22

**Authors:** Yassin Nouar, Dia Eddine Oudghiri, Adil Najdi, Nisrin El Mlili

**Affiliations:** 1 Nursing, Faculty of Medicine, Abdelmalek Essaâdi University, Tangier, MAR; 2 Nursing, Higher Institute of Nursing Professions and Health Techniques of Tetouan, Tetouan, MAR; 3 Biology and Health, Faculty of Sciences, Abdelmalek Essaâdi University, Tetouan, MAR; 4 Laboratory of Epidemiology and Public Health, Mohamed VI University Hospital of Tangier, Tangier, MAR; 5 Health Sciences, Higher Institute of Nursing Professions and Health Techniques of Tetouan, Tetouan, MAR

**Keywords:** northern morocco, quality of life, irregular, sub-saharan, migrants

## Abstract

Introduction: Due to its geographical proximity to Europe, Morocco experiences a significant influx of migrants from neighboring Sub-Saharan African countries. Attempts to cross the Strait of Gibraltar make Northern Morocco a stopover for Sub-Saharan migrants. However, faced with increasingly restrictive measures from European Union countries, many of these migrants are compelled to turn this transit into a prolonged stay in Morocco, often in irregular status and facing challenging socio-economic conditions that threaten their health and their quality of life-related to health. This study aims to assess the level of health-related quality of life and its determinants among irregular Sub-Saharan migrants in Northern Morocco using the 36-Item Short Form Survey (SF-36) measurement scale.

Method: A total of 526 irregular Sub-Saharan migrants residing in the cities of Tangier and Tetouan were recruited using snowball sampling. A socio-demographic information sheet and the SF-36 scale were used to collect data. Subsequently, the data were entered and statistically analyzed using Statistical Product and Service Solutions (SPSS, version 21.0; IBM SPSS Statistics for Windows, Armonk, NY). Numerical variables were summarized as mean ± standard deviation and categorical variables as frequency and percentage. Non-parametric tests, including the Mann-Whitney U test and Kendall's Tau-B, were used to measure the association of physical and mental component scores with sociodemographic variables, for a significance level set at p < 0.05. Multivariate logistic regression was conducted to identify factors determining health-related quality of life, using odds ratios (OR) and a 95% confidence interval (CI) for p < 0.05.

Results: Participants were aged between 18 and 50 years, with a mean age of 29.7 ± 7.6 years; 80% were single, and the majority were male (63.7%). In terms of education, 57.2% have a secondary level. The physical (PCS = 46.5 ± 9.03) and mental (MCS = 35.5 ± 9.9) summary scores were low. PCS and MCS were lower among subjects aged ≥ 36 years compared to those aged 18-25 years (p = 0.000). Women had lower PCS than men (p = 0.002). The migrants having more than three years in Morocco had lower MCS compared to those recently settled (p = 0.002). PCS and MCS were lower among participants who were assaulted compared to those who were not (p = 0.000). Multivariate logistic regression results showed that age, type of habitat, and assault significantly affected both PCS and MCS scores. Gender is a significant determinant for PCS and duration of stay in Morocco for MCS.

Conclusion: The quality of life of Sub-Saharan irregular migrants appears to be low, particularly concerning the mental health component, highlighting the need for priority interventions in this area to improve their health status.

## Introduction

Migration is a phenomenon as old as humanity itself. For millennia, human beings have left their place of birth to settle elsewhere, whether for economic, political, social, or environmental reasons. Migration has an influence on the physical, mental, and emotional health and well-being of migrants [1]. It brings about changes in people's lives such as social relationships, climate, language, culture, and diet. Indeed, migrants are often required to adapt to a new social and cultural environment that is often unrelated to their prior world [2].

In general, the migration process has frequently been associated with challenging experiences and, consequently, greater vulnerability to health issues and a decline in quality of life (QOL) [3]. Living in unsanitary housing and having low incomes can lead to a deterioration in QOL [4].

Given the rapid growth of global mobility, migrant health has become an important field in medical science [5]. Research conducted worldwide has shown a decrease in health-related QOL (HRQOL) among migrant populations compared to the native population [6]. Thus, maintaining good health and optimal QOL can prove very challenging for many migrants.

Measuring the health status of a population extends beyond traditional measures of morbidity and mortality to include the assessment of QOL. QOL is a multidimensional concept that includes critical aspects, such as physical health, psychological well-being, social relationships, economic situation, personal beliefs, and the connections of these aspects with the environment. HRQOL can also refer to subjective well-being related to health, which is the self-assessed health status of the individual themselves [7].

HRQOL depends on many factors, such as health status, employment, education, access to healthcare, culture, and social status [8]. It has been found that measuring HRQOL is considered a rapid and effective way to assess health status, taking into account socio-economic determinants [9].

Several tools have been used in the literature to assess the QOL related to health, for example, The Quality of Well-being Scale (QWB) [10] and The Duke Health Profile [11]. The 36-Item Short Form Survey (SF-36) questionnaire is one of the most commonly used generic tools to measure HRQOL in both clinical practice and scientific research due to its brevity, high reproducibility, validity, and sensitivity to changes. It allows for the assessment and monitoring of the health status of a given population. Indeed, it is a self-administered questionnaire that generates scores reflecting perceived health status [12].

The SF-36 has been translated into more than 50 languages. Its psychometric properties have been demonstrated across various pathological conditions and among different populations, including some non-Western cultures and ethnic minorities in certain countries. It is currently considered the "gold standard" for assessing HRQOL [13]. It is also particularly well-suited and advantageous for use in large-scale health assessments (N = 500+) and in situations requiring summary information on both physical and mental health status.

In northwest Italy, particularly in the city of Genoa, migrants have moderate levels of HRQOL, with the mental component significantly lower than the physical component. It has been observed that lower scores in the mental dimension of HRQOL were associated with clinical depression [14].

According to a Spanish study on Moroccan immigrants settled in the Basque country, dimensions of HRQOL reveal early symptoms of anxiety and depression. Spaniards exhibit higher HRQOL scores compared to Moroccan immigrants, but differences appear to strongly depend on social support variables [15]. Migrants in Spain have been reported to have a lower HRQOL than native Spaniards, which is linked to their disadvantages in terms of socioeconomic status, social support, and psychological distress [16].

Data from a study conducted on foreigners in Belgium revealed that two-thirds of the interviewees suffer from at least one chronic disease, and one-third suffer from a diagnosed mental health problem, although it is likely that this proportion is actually higher because many terms used by the individuals interviewed refer to sleep disorders, anxiety, or stress [17].

Although the mental component of HRQOL among Mexican-origin Americans living in settlements is similar to that of the general U.S. population, the physical health component is markedly poorer. Low education levels and long-term residency in the settlements were predictive factors of poorer physical health [18].

In Morocco, given its geographical proximity to Spain and the advent of the "New Moroccan Migration Policy" in 2013, an increasing number of migrants, primarily from neighboring Sub-Saharan African countries, are making Morocco a transit country to reach Europe or even a final destination [19]. According to the Ministry Delegate in charge of Moroccans Residing Abroad and Migration Affairs, approximately 54.000 migrants were regularized through the two regularization operations conducted in 2014-2015 and 2017-2018.

In 2021, the High Commission for Planning in Morocco (HCP) conducted a national survey covering a sample of 3.000 migrants aged ≥ 15 years, 71.6% (N=3000) of them reported being in an irregular situation.

It is worth noting the absence of reliable and current statistics on the number of individuals in irregular situations in Moroccan territory. According to organizations working to assist immigrants in vulnerable situations, it is in the northern part of the country where most irregular immigrants are found (National Strategic Plan for Health and Immigration 2021-2025).

In this study, we aimed to assess the level of HRQOL and its determinants among irregular Sub-Saharan migrants in northern Morocco using the SF-36 measurement scale.

Understanding HRQOL is essential for following the health status of vulnerable populations such as migrants, aiming to reduce healthcare disparities and develop effective interventions to improve or preserve QOL.

## Materials and methods

Study design and participants

Due to the absence of a pre-established list of details about the target population and the particular undocumented status of the participants, snowball sampling was used, which is defined as a non-probabilistic sampling method. 

This sampling technique allows the researcher to initially contact a few participants who can then refer others within their networks, and the enrollment for this cross-sectional study took place between September 2022 and February 2023. 

To be eligible for inclusion, subjects had to be of Sub-Saharan African origin, aged ≥ 18 years, not have Moroccan nationality, not possess a residence permit, have sufficient knowledge of French, English, or Spanish to fill out the questionnaire (for illiterate participants, the questionnaire was filled out by the investigator who interviewed the migrant), and reside in the cities of Tetouan or Tangier. The initial participants were recruited from social promotion associations ("Helping Hands" and "Organization of Young Africans"). The sample size for our study was N=526 irregular Sub-Saharan migrants (N=335 men, representing 63.7% of the sample, and N=191 women, with a percentage of 36.3%). These migrants are distributed between the city of Tetouan (128) and the city of Tangier (398).

Data collection

We used an information sheet to collect sociodemographic data, as well as data about participants' personal safety (whether they have been victims of aggression) and the attitudes and behaviors of Moroccans towards migrants, and we used the SF-36 to assess their quality of life. Depending on the native language of the participant, the French [20], English [21], or Spanish version of SF-36 [22] was used.

The SF-36 consists of 36 questions grouped into eight dimensions of perceived health: physical functioning (PF), role physical (RP), bodily pain (BP), general health (GH), vitality (VT), social functioning (SF), mental health (MH), and role emotional (RE). The first four dimensions can be summarized into a physical component summary score (PCS), while the latter four are from a mental component summary (MCS) score. Each dimension and summary score ranges from zero to 100, where a high score indicates a high level of activity and/or good health, whereas a low score indicates a low level of activity and/or poor health [23].

Overall, we analyzed 10 scores in this study: the scores from the eight domains of the SF-36 plus the two summary scores for physical and mental health. 

Statistical analysis

Statistical Product and Service Solutions (SPSS, version 21.0; IBM SPSS Statistics for Windows, Armonk, NY) software was used to analyze data. Descriptive statistics were calculated for all variables. Quantitative variables were summarized as mean ± standard deviation (SD), while qualitative variables were presented as frequencies and percentages. 

To assess the association of the physical component summary (PCS) and MCS scores with sociodemographic variables, non-parametric tests were conducted: the Mann-Whitney U test for independent samples was used to compare means between the two groups. Meanwhile, Kendall's Tau-B was used to compare means among three or more groups. The significance level adopted was set at p < 0.05. 

To predict the determinants of HRQOL, multivariate logistic regression analysis was performed for PCS and MCS scores. Measures of relative effect were expressed as odds ratios (OR) with 95% confidence intervals (CI). The significance level was set at p < 0.05.

Ethical considerations

Participants signed the informed consent and anonymity was protected. The Ethics Committee for Biomedical Research of Oujda (Faculty of Medicine and Pharmacy, Mohammed First University, Oujda, Morocco) approved the study protocol (Protocol No. 01/2022).

## Results

Sociodemographic characteristics of participants

A total of 526 irregular Sub-Saharan migrants responded to the questionnaire. The age ranged from 18 to 50 years with a mean of 29.7±7.6 years (Table 1). 

**Table 1:** Distribution of sociodemographic characteristics of irregular Sub-Saharan migrants living in northern Morocco. Data are presented as mean ± standard, numbers, and percentages.

Variable		Mean (Standard Deviation)	Numbers	Percentage (%)
Sex	Male	-	335	63.7
	Female	-	191	36.3
Age (years)	18-25	29.7 (7.6)	196	37.2
	26-35		195	37
	≥ 36		135	25.8
Marital Status	Single	-	421	80
	Married	-	67	12.8
	Divorced	-	26	5
	Widowed	-	12	2.2
Education Level	Illiterate	-	55	10.5
	Primary	-	105	20
	Secondary	-	301	57.2
	Higher	-	65	12.3
Type of Housing	New town	-	127	24.2
	Old town	-	162	30.7
	Precarious Housing	-	179	34
	Rural Housing	-	58	11.1
Physical Activity Practice	Yes	-	206	39.2
	No	-	320	60.8
Length of Stay in Morocco	< 2 years	-	137	26
	De 2 à 3 years	-	160	30.5
	> 3 years	-	229	43.5
Victim of Aggression	Yes	-	397	75.4
	No	-	129	24.6
Type of Aggressions	Verbal	-	206	51.9
	Physical	-	185	46.6
	Sexual	-	6	1.5

Further categorization of age revealed that about three-quarters of the participants were aged between 18 and 35 years, with 37.2% (N=526) aged 18-25 years and 37% (N=526) aged 26-35 years, 80% (N=526) of the respondents being single, and the majority were men (63.7%, N=526). In terms of education, 57.2% (N=526) reported having secondary education, while illiterate individuals constituted the lowest percentage at 10.5% (N=526).

Nearly three-quarters of the surveyed migrants, or 74% (N=526), had been living in Morocco for more than two years, with 43.5% (N=526) having resided there for over three years. Two-thirds (64.7%, N=526) lived either in old houses (old city) or in precarious peri-urban habitats. Only one-third of the participants engaged in physical activity, particularly walking and occasionally football in groups formed by migrants. Additionally, 75.4% (N=526) reported having been victims of aggression, including verbal (51.9%, N=397), physical (46.6%, N=397), and rarely sexual (1.5%, N=397) assaults, with the majority of these aggressions (97.8%, N=397) occurring in Morocco, mainly in large cities.

Average scores of HRQOL

**Figure 1:**
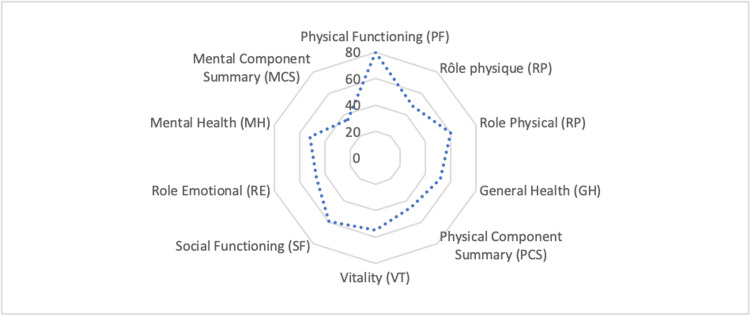
Distribution of the average quality of life scores among irregular Sub-Saharan migrants living in northern Morocco.

The physical and mental summary scores obtained among the irregular Sub-Saharan migrant population are relatively low. However, the PCS of 46.5 ± 9.03 is higher than the mental summary score of 35.5 ± 9.9 (Figure 1).

The overall sample exhibits a relatively high physical functioning score (PF=79.9 ± 23.02). However, participants experienced significant limitations due to physical health (RP=48.4 ± 39.9). Additionally, bodily pain scores were notable (BP=60.2 ± 26.07). The general health status of the studied subjects is considered average (GH=51.9 ± 20.7).

The vitality score is fairly average (VT=54.6 ± 17.3), reflecting a sense of fatigue and lack of energy among the participants. Migrants also experience moderate difficulties in social and interpersonal relationships, as indicated by a slightly above-average score (SF=59.2 ± 18.3). Additionally, they report that emotional problems significantly limit their regular daily activities, as evidenced by a below-average score in role emotional (RE=46.5 ± 41.6). Thus, the mental health of individuals in our population is average (MH=52.1 ± 17.4).

Association between respondents’ sociodemographic characteristics and their HRQOL

The results showed that age was significantly associated with all subscales measuring quality of life. Scores for PF, MH, and GH decreased with increasing age (p=0.000). Conversely, the youngest age group (18-25 years) in our sample had higher scores for bodily pain (BP=70.4 ± 26.2) compared to the oldest age group (≥ 36 years) with a score of BP=53.2 ± 23.7 (p=0.000) (Table 2).

**Table 2:** Association between SF-36 scores and sociodemographic characteristics (sex, age, physical activity, marital status, and education level) of irregular Sub-Saharan migrants living in northern Morocco. Data (N=526) are presented as mean ± standard deviation. *The correlation is significant at p < 0.05​​.

SF 36 Dimensions	Sex		P value	Age Groups (years)			P-value	Physical Activity Practice		P-value
	Male Mean (SD)	Female Mean (SD)		18-25 Mean (SD)	26-35 Mean (SD)	≥ 36 Mean (SD)		Yes Mean (SD)	No Mean (SD)	
Physical Functioning (PF)	82.5 (22.5)	75.5 (23.2)	0.000*	84.9 (19.6)	78.01 (23.8)	75.1 (25.3)	0.000*	84.1 (20.8)	77.3 (23,1)	0.000*
Role Physical (RP)	48.7 (39.6)	48.01 (40.5)	0.854	61.7 (38.5)	44.8 (38.4)	38.3 (33.2)	0.000*	53.2 (39.01)	43.2 (39,4)	0.005*
Bodily Pain (BP)	62.2 (25.6)	56.8 (26.4)	0.015*	70.4 (26.2)	54.3 (24.05)	53.2 (23.7)	0.000*	66.08 (25.4)	57.3 (25,5)	0.000*
General Health (GH)	53.4 (20.3)	49.2 (21.2)	0.04*	59.5 (19.3)	49.06 (21.1)	44.6 (18.3)	0.000*	56.8 (20.2)	47.1 (20,2)	0.000*
Vitality (VT)	56.02 (17.4)	52.1 (16.8)	0.028*	59.9 (16.4)	52.7 (17.1)	49.9 (15.8)	0.000*	59.4 (17)	50.7 (16,1)	0.000*
Social Functioning (SF)	60 (18.6)	57.8 (17.6)	0.21	64.4 (18.7)	56.1 (17.9)	55.7 (16.5)	0.000*	61.2 (19.2)	56.4 (16,03)	0.009*
Role Emotional (RE)	46.3 (41.6)	47.01 (41.7)	0.85	57.3 (41.5)	43.5 (40.9)	39.1 (34.1)	0.000*	50.3 (41.7)	42.6 (41,2)	0.04*
Mental Health (MH)	52.1 (17.9)	52.1 (16.4)	0.872	57.3 (18.0)	50.2 (16.7)	48.03 (15.6)	0.000*	53.9 (16.6)	49.7 (16,3)	0.003*
Physical Component Summary (PCS)	47.4 (8.6)	44.8 (9.4)	0.002*	49.6 (7.7)	45.1 (8.8)	43.6 (9.7)	0.000*	48.7 (8.1)	45.01 (9,3)	0.000*
Mental Component Summary (MCS)	35.3 (10.1)	35.8 (9.4)	0.485	38.2 (9.4)	34.6 (10.1)	32.9 (9.3)	0.000*	36.4 (9.9)	34.3 (9,2)	0.02*
SF 36 Dimensions	Marital Status				P value	Level of education				P value
	Single Mean (SD)	Married Mean (SD)	Divorced Mean (SD)	Widowed Mean (SD)		Illiterate Mean (SD)	Primary Mean (SD)	Secondary Mean (SD)	Higher Mean (SD)	
Physical Functioning (PF)	81.5 (21.1)	78.1 (27.6)	69.8 (26.7)	57.08 (32.2)	0.028*	80 (20.7)	80.3 (22.6)	79.6 (23.2)	81.2 (23.9)	0.550
Role Physical (RP)	49.4 (39.2)	42.5 (44.2)	52.8 (41.4)	33.3 (32.5)	0.180	53.6 (38.9)	52.9 (39.4)	45.1 (40.07)	52.3 (40.1)	0.254
Bodily Pain (BP)	61.5 (26.4)	56.4 (21.8)	53.8 (25.6)	45.9 (30.5)	0.011*	61.5 (23.4)	57.1 (26.1)	60.6 (26.8)	61.7 (24.5)	0.675
General Health (GH)	52.2 (20.6)	54.5 (20.4)	46.5 (20.8)	36.1 (18.2)	0.256	49.6 (20.1)	51.8 (21.4)	51.6 (20.8)	54.8 (20.5)	0.350
Vitality (VT)	54.64 (17.2)	57.5 (19.1)	51.1 (12.6)	42.7 (12.7)	0.425	52.2 (15.8)	51.8 (15.1)	54.4 (18.01)	61.2 (16.5)	0.000*
Social Functioning (SF)	59.01 (18.01)	61.5 (21.5)	54.8 (16.2)	60.2 (10.9)	0.630	57.7 (19.01)	60.5 (17.3)	58.4 (18.7)	61.9 (17.1)	0.545
Role Emotional (RE)	47.6 (41.2)	40 (44.5)	51.2 (41.3)	38.8 (39.7)	0.286	53.9 (38.7)	55.2 (40.2)	40.9 (41.5)	52.05 (43.6)	0.059
Mental Health (MH)	52 (17.2)	54.6 (20.6)	51.3 (11.7)	42.1 (10.6)	0.547	47.1 (15.6)	51.6 (15.8)	52.02 (17.9)	57.8 (17.5)	0.002*
Physical Component Summary (PCS)	47.02 (8.5)	45.7 (9.6)	43.3 (11.5)	38.2 (10.9)	0.007*	46.8 (8.8)	46.01 (9.2)	46.5 (8.9)	46.6 (9.4)	0.805
Mental Component Summary (MCS)	35.4 (9.7)	35.9 (12.2)	36.5 (8.2)	34.03 (6.6)	0.841	34.9 (8.6)	36.6 (9.4)	34.6 (10.1)	38.6 (10.07)	0.403

The results also showed that the group of migrants aged ≥ 36 years had lower vitality scores (VT=49.9 ± 15.8) and moderate scores for social functioning (SF=55.7 ± 16.5), which explains the limitations in their daily activities due to their emotional states (RE=39.1 ± 34.1, the lowest compared to other age groups; p=0.000). Overall, the physical and mental summary scores for subjects aged ≥ 36 years (PCS=43.6 ± 9.7 and MCS=32.9 ± 9.3) were lower than those for subjects aged 18-25 years (PCS=49.6 ± 7.7 and MCS=38.2 ± 9.4; p=0.000) (Table 2).

Physical and mental summary scores were higher among sports participants (PCS=48.7 ± 8.1 and MCS=36.4 ± 9.9) compared to non-sports participants (PCS=45.01 ± 9.3, p=0.000 and MCS=34.3 ± 9.2, p=0.02) (Table 2).

Women had significantly lower scores than men in both physical components PF (PF=75.5 ± 23.2 versus 82.5 ± 22.5; p=0.000) and GH (GH=49.2 ± 21.2 versus 53.4 ± 20.3; p=0.04). Additionally, the PCS score was lower for women (p=0.002). Women reported feeling more tired and having less energy than men, with vitality scores of VT=52.1 ± 16.8 for women and VT=56.02 ± 17.4 for men (p=0.028). Regarding marital status, it was found that unmarried individuals had statistically significant higher scores in PF (p=0.028), BP (p=0.011), and PCS (p=0.007) (Table 2).

MH was significantly associated with education level (p=0.002), where MH scores increased proportionally with higher levels of education (Table 2).

The results also showed that only the MCS was associated with the length of stay in Morocco (p=0.002); migrants with more than three years of stay had lower scores (MCS=34.7 ± 10.2) compared to those who recently settled (MCS=38.3 ± 9.4). Additionally, QOL varied by type of housing; individuals living in new city areas had lower scores in several physical components: PF=73.2 ± 24.3 (p=0.000), GH=48.7 ± 23.2 (p=0.038), and PCS=42.8 ± 10.7 (p=0.000). However, participants living in precarious habitats had lower scores in both mental dimensions, MH=49.3 ± 16.2 (p=0.002) and MCS=33.1 ± 10.4 (p=0.000). For those settled in rural areas (forest), they reported experiencing physical pain (BP=66.9 ± 21.9, p=0.000) (Table 3).

**Table 3:** Association between SF-36 scores and socio-demographic characteristics (Length of stay in Morocco and type of housing) of irregular Sub-Saharan migrants living in northern Morocco. Data (N=526) are presented as mean ± standard deviation. *The correlation is significant at p < 0.05.

SF 36 Dimensions	Length of Stay in Morocco			P value	Type of Housing				P value
	< 2 years mean (SD)	2-3 years mean (SD)	> 3 years mean (SD)		New town mean (SD)	Old town mean (SD)	Precarious housing mean (SD)	Rural housing mean (SD)	
Physical Functioning (PF)	78.5 (22.9)	80.08 (22.5)	80.8 (23.4)	0.208	73.2 (24.3)	77.8 (24.5)	85.4 (19.3)	84.2 (16.5)	0.000*
Role Physical (RP)	50.5 (39.1)	46.5 (38.9)	48.4 (41.1)	0.528	45.4 (39.6)	41.1 (39.01)	50.3 (39.2)	56.6 (39.8)	0.043*
Bodily Pain (BP)	60.4 (27.7)	59.8 (24.4)	60.4 (26.1)	0.743	52.9 (28.7)	59.2 (25.01)	65.5 (23.7)	66.9 (21.9)	0.000*
General Health (GH)	52.1 (20.5)	50.6 (19.9)	52.5 (21.4)	0.941	48.7 (23.2)	49.4 (22.4)	52.7 (17.4)	54.2 (18.8)	0.038*
Vitality (VT)	55.3 (15.88)	52.8 (17.01)	55.2 (18.3)	0.651	55.3 (15.2)	53.1 (17.04)	54.2 (18.4)	54.1 (16.09)	0.647
Social Functioning (SF)	61.7 (18.3)	57.5 (15.9)	58.7 (19.6)	0.147	58.8 (16.6)	58.07 (18.8)	56.3 (17.08)	63.9 (15.5)	0.430
Role Emotional (RE)	56.5 (39.1)	41.7 (41.08)	43.4 (42.6)	0.08	50 (39.9)	40.4 (40.7)	44.2 (43.6)	53.1 (39.9)	0.973
Mental Health (MH)	54.4 (16.5)	50.3 (16.01)	51,8 (18.7)	0.292	57.7 (16.4)	49.6 (16.7)	49.3 (16.2)	49.7 (12.6)	0.002*
Physical Component Summary (PCS)	45.5 (8.8)	46.6 (8.8)	47.06 (9.2)	0.124	42.8 (10.7)	45.6 (8.7)	48.9 (7.4)	49.09 (7.2)	0.000*
Mental Component Summary (MCS)	38.3 (9.4)	34.09 (9.4)	34.7 (10.2)	0.002*	38.9 (8.4)	34.3 (8.8)	33.1 (10.4)	35.6 (8.2)	0.000*

Sub-Saharan irregular migrants in our sample reported having been victims of aggression during their migration experiences. Significant differences were observed in the QOL dimensions between participants who experienced aggression versus those who did not, for (PCS=45.5 ± 9.03 versus 49.2 ± 8.7; p=0.000) and (MCS=33.6 ± 9.08 versus 39.6 ± 9.7; p=0.000).

Regarding types of aggression, respondents who experienced physical aggression showed lower scores in physical functioning (PF=74.3 ± 22.01, p=0.000) and MH (46.02 ± 14.3, p=0.011) compared to other types of aggression. Those who reported verbal aggression had a lower mental summary score (MCS=32.5 ± 9.3, p=0.004) and reported higher than average physical pain (BP=60.07 ± 20.6, p=0.004) (Table 4).

The results revealed a low number of sexual assaults (six cases); victims of these assaults showed lower general health scores (GH=44.3 ± 26.2, p=0.013) and lower physical summary scores (PCS=42.8 ± 10.2, p=0.000). However, these victims did not report significant limitations in daily activities due to emotional issues (RE=66.6 ± 42.1, p=0.011) (Table 4).

**Table 4:** Association between SF-36 scores and violence (victim of aggression and its types) among irregular Sub-Saharan migrants living in northern Morocco. Data (N=526) are presented as mean ± standard deviation. *The correlation is significant at p < 0.05.

SF 36 Dimensions	Victim of Aggression		P value	Type of Aggressions			P value
	Yes (Mean ± SD)	No (Mean ± SD)		Verbal (Mean ± SD)	Physical (Mean ± SD)	Sexual (Mean ± SD)	
Physical Functioning (PF)	79.03 (22.6)	82.8 (22.1)	0.043*	83.03 (22.6)	74.3 (22.01)	89.1 (12.8)	0.000*
Role Physical (RP)	41.5 (38.1)	64.3 (39.02)	0.000*	45.2 (40.6)	37.5 (35.06)	41.6 (34.1)	0.197
Bodily Pain (BP)	56.7 (25.2)	72.9 (23.6)	0.000*	60.07 (20.6)	53.7 (28.7)	41.1 (37.5)	0.004*
General Health (GH)	48.3 (20.6)	59.1 (19.1)	0.000*	50.4 (16.2)	46.1 (24.2)	44.3 (26.2)	0.013*
Vitality (VT)	51.4 (15.8)	62.1 (18)	0.000*	51.9 (15.3)	50.8 (16.4)	55 (14.1)	0.587
Social Functioning (SF)	56.2 (16.4)	64.6 (19.3)	0.000*	54.3 (14.5)	58.2 (18.1)	58.3 (10.2)	0.061
Role Emotional (RE)	41.3 (40.3)	58.6 (42.8)	0.000*	37.1 (42.5)	44.9 (37.2)	66.6 (42.1)	0.011*
Mental Health (MH)	48.2 (14.6)	60.9 (18.5)	0.000*	50.3 (14.6)	46.02 (14.3)	49.3 (14)	0.011*
Physical Component Summary (PCS)	45.5 (9.03)	49.2 (8.7)	0.000*	47.3 (8.08)	43.6 (9.6)	42.8 (10.2)	0.000*
Mental Component Summary (MCS)	33.6 (9.08)	39.6 (9.7)	0.000*	32.5 (9.3)	34.7 (8.6)	38.8 (8.5)	0.004*

To identify the determinants of HRQOL, a multivariate logistic regression analysis was conducted. As shown in Table 5, the results indicated that age, type of habitat, and aggression significantly impacted both physical (PCS) and mental (MCS) summary scores. However, gender was found to be a significant determinant only for PCS, whereas length of stay in Morocco was significant only for MCS.

**Table 5:** Determinants of the quality of life of irregular Sub-Saharan migrants living in northern Morocco. N=526. *The correlation is significant at p < 0.05.

Variable		Physical Component Summary (PCS)		Mental Component Summary (MCS)	
		Odds ratio (IC 95%)	P value	Odds-ratio (IC 95%)	P value
Sex	Female	1.0 (reference)	-	-	-
	Male	1.782 (1.138-2.792)	0.012	-	-
Age group	≤ 25 years	1.0 (reference)	-	1.0 (reference)	-
	26-35 years	0.490 (0.303-0.795)	0.004	0.502 (0.316-0.8)	0.004
	≥ 36 years	0.286 (0.164-0.498)	0.000	0.449 (0.265-0.762)	0.003
Type of Housing	New town	1.0 (reference)	-	1.0 (reference)	-
	Old town	-	-	0.386 (0.223-0.668)	0.001
	Precarious Housing	2.889 (1.642- 5.083)	0.000	0.351 (0.203-0.605)	0.000
	Rural Housing	2.334 (1.077-5.057)	0.032	-	-
Length of Stay in Morocco	< 2 years	-	-	1.0 (reference)	-
	> 3 years	-	-	0.561 (0.341-0.923)	0.023
Aggression	No Aggression	1.0 (reference)	-	1.0 (reference)	-
	Verbal Aggression	-	-	0.453 (0.271-0.759)	0.003
	Physical Aggression	0.273 (0.157-0.475)	0.000	-	-

Male migrants had an odds ratio (OR) of 1.782 (95% CI: 1.138-2.792) for PCS, indicating a higher likelihood of having a higher PCS score compared to females (p=0.012).

Compared to individuals aged 25 years or younger (reference category), those aged 26-35 years had an OR of 0.490 (95% CI: 0.303-0.795) for PCS (p=0.004) and 0.502 (95% CI: 0.316-0.800) for MCS (p=0.004), indicating significantly lower odds of having high scores in these components. Similarly, individuals aged 36 years or older showed an OR of 0.286 (95% CI: 0.164-0.498) for PCS (p=0.000) and 0.449 (95% CI: 0.265-0.762) for MCS (p=0.003), suggesting an even greater decrease in the likelihood of having high scores compared to younger individuals (≤ 25 years). Thus, advanced age is considered a determinant of poorer HRQOL.

Migrants living in the old city and in precarious habitats had OR of 0.386 (95% CI: 0.223-0.668) with (p=0.001) and 0.351 (95% CI: 0.203-0.605) with (p=0.000) for mental component scores (MCS), respectively, indicating lower mental summary scores compared to those residing in the new city (reference category). However, residents of precarious and rural areas had significantly higher OR of 2.889 (95% CI: 1.642-5.083) with (p=0.000) and 2.334 (95% CI: 1.077-5.057) with (p=0.032) for physical component scores (PCS), respectively. These two categories thus had a higher probability of obtaining higher physical scores compared to residents of the new city (reference category). Participants who had been in Morocco for more than three years had an OR of 0.561 (95% CI: 0.341-0.923) for MCS (p=0.023), indicating lower mental scores compared to those who had settled recently (< 2 years). 

Referring to migrants who were not victims of violence, lower scores in physical dimension (PCS) were associated with physical aggression (OR= 0.273, 95% CI: 0.157-0.475 with p=0.000), and lower mental scores (MCS) were associated with verbal aggression (OR=0.453, 95% CI: 0.271-0.759 with p=0.003) among those who were victims of aggression.

## Discussion

The result obtained from a sample of 526 irregular Sub-Saharan migrants settled in northern Morocco highlighted several aspects of their sociodemographic profile and experiences. These migrants come from 25 countries, including 51% (N=264) from the West African region, 44% (N=230) from Central Africa, and 5% (N=24) from East Africa. This population was predominantly composed of young adults, with an average age of 29.79 ± 7.6 years, and the 18-35 age group was the most predominant, reflecting the youthfulness of this particular demographic group. In terms of gender, males constituted the majority. Educationally, more than half (57.2%, N=526) had a secondary level of education. Notably, 80% (N=526) of respondents were single, suggesting a trend where young singles may be more inclined to undertake risky migration journeys.

The youthfulness of the studied population suggests that factors such as unemployment and limited economic opportunities in their countries of origin may influence their decision to migrate in pursuit of a better life [24]. 

This migratory process results from a combination of three types of factors: firstly, there are generative factors such as the unemployment rate (the proportion of unemployed individuals among the active population aged 15 years and above in Sub-Saharan Africa is 5.8% (N=470,611,000), according to the ILO's modeled estimates for 2023), the poverty rate (37.2% (N=1,241,897,820) of the population in Sub-Saharan Africa lives below the poverty line, World Bank 2023), high demographic growth (4.6 births per woman, United Nations 2021), recurring years of drought, and political conflicts across Africa. In addition, there are incentive factors such as the image of social success projected by immigrants who return to their home countries during annual vacations, as well as the portrayal of Western lifestyles through audiovisual media. Finally, there are pull factors in the host countries characterized by an excess demand for unskilled labor and the creation of structured networks for trafficking migrants. This trafficking has become a more profitable and less risky business than drug trafficking with traffickers' profits amounting to $10 billion annually [25]. All these factors particularly drive young Africans to look towards the North and consider North Africa as a transit and passage destination towards the European dream.

Indeed, faced with increasingly restrictive measures from EU countries and the sophisticated logistics of the Integrated System of External Surveillance (SIVE), these migrants are forced to prolong their stay in Morocco. Currently, the average stay ranges from nine months to two years [25]. In our sample, nearly three-quarters of migrants (74%, N=526) have stayed in Morocco for more than two years, indicating that their transit situation has turned into a residency or settlement situation, requiring integration and adaptation to a new way of life [2]. Consequently, the European dream becomes even more difficult to achieve, generating feelings of hopelessness and frustration among these migrants.

The living conditions of this migrant population are precarious, with a significant proportion (64.7%, N=526) residing in old housing or peri-urban slums, which could influence their well-being and daily security. This observation aligns with findings by Khachani in 2006 [25].

A concerning aspect of the study involves high levels of aggression experienced by migrants, with 75.4% (N=526) reporting being victims of various types of aggression: verbal (51.9%, N=397), physical (46.6%, N=397), and rarely sexual (1.5%, N=397). These incidents mainly occurred in major Moroccan cities, highlighting the ongoing challenges faced by Sub-Saharan migrants in terms of personal safety and social integration during their migration journey, including exploitation and physical dangers. These findings are consistent with a study conducted by Leyva-Flores et al. [26], among migrants transiting through Mexico to the United States between 2009 and 2015, which showed an overall prevalence of violence in all forms at 29.4% (N=12.023). Approximately 24% (N=3,535) reported experiencing physical violence, 19.5%(N=3,535) psychological violence, and around 2% (N=3,535) sexual violence [26]. These aggressions can be attributed to the irregular migration status of the majority of these migrants, who seek to escape rather than report incidents to authorities due to their lack of legal documentation. Consequently, they prefer to take routes less monitored by authorities, which unfortunately are also frequented by criminal organizations.

The results from the assessment of summary scores of physical and mental health among irregular Sub-Saharan migrant populations indicate an overall perception of lower-than-average physical and mental QOL. The scores are relatively low, with an average of PCS=46.5±9.03 and MCS=35.5±9.9. This finding is consistent with that observed in a sample of 200 undocumented migrants in Rome in 2014, where the scores were PCS=46.5 and MCS=37.9, respectively [24].

Comparing the PCS and MCS scores of our study population to those obtained from a sample of 385 individuals living in Tetouan, northern Morocco (PCS=45.8±8.4 and MCS=40.9±11.4) [23], we observe that migrants in our study have 0.7% higher PCS scores and 3% lower MCS scores. Undocumented status, cultural conflict (acculturation or deculturation), socio-economic conditions of migrants, as well as the gap between aspirations and achievements, are factors of vulnerability and stress that influence individuals' mental state [3].

The results of the physical health scores (RP = 48.4 ± 39.9), bodily pain (BP=60.2 ± 26.07), and general health (GH=51.9 ± 20.7) observed in this study show that participants experience physical health issues that impact their ability to lead a normal and active life. Disadvantaged migrants often report frequent headaches, previous injuries, and accidents [5,6].

Furthermore, vitality scores (VT=54.6 ± 17.3), social functioning (SF=59.2 ± 18.3), emotional role (RE=46.5 ± 41.6), and mental health (MH=52.1 ± 17.4) highlight the emotional issues faced by migrants, thus limiting their usual daily activities. Sleep disturbances and anxiety are symptoms related to stress due to the migration process, living conditions, and other environmental and personal factors [1].

The analysis of associations between sociodemographic variables and HRQOL revealed correlations between summary scores for physical and mental components and certain sociodemographic determinants among participants, namely: gender, age, type of habitat, duration of stay in Morocco, and aggression.

Firstly, age appears as a significant determinant for both physical (PCS) and mental (MCS) summary scores. Compared to young adults (aged ≤ 25 years) used as a reference group, migrants aged 26-35 years have a significantly lower probability of achieving high scores for PCS and MCS.

The probability decreases further for migrants aged ≥ 36 years, suggesting a progressive decline in HRQOL with age. Several studies have shown that SF-36 scores decrease with age, including studies such as Nesterko et al.'s study on HRQOL and life satisfaction among immigrants and native Germans [27], and El Emrani et al.'s study measuring the influence of gender and age on HRQOL in the population of Tetouan, Morocco [22].

Young adults aged 18-25 years in our sample recorded a low MCS score (38.2 ± 9.4). This score can be explained by the characteristics of this age group, which relate to subjective feelings about reaching adulthood, specifically the two main criteria: personal responsibility and the ability to make independent decisions. A third, more tangible criterion is becoming financially independent [28]. Regarding psychological and emotional aspects, most young adults perceive the process of becoming an adult as a loss of the "safety net" that their parents provided during adolescence [29]. Research suggests that the two most prevalent mental health issues among first-generation young immigrants are major depression and anxiety disorders, which can be triggered by stressful events such as family separation, exposure to discrimination, traumatic events, and others [14].

The results indicate that gender is significantly associated only with the PCS score, with men having a higher probability of having higher physical scores than women. This finding is consistent with studies conducted by El Emrani et al. in northern Morocco in 2016 [23], Domnich et al. in northwest Italy in 2010 [14], and Daher et al. in Malaysia in 2010 [6]. This gender difference may suggest that women have a more meticulous perception of their medical issues, especially minor ones, than men, and therefore they are more likely to report them [14]. The lower score among women may also be explained by hormonal variations, childbirth, and the roles of motherhood and wifehood, which are often detrimental to a woman's health [6].

Migrants who have resided in Morocco for more than three years show significantly lower MCS scores compared to those who have settled more recently (< two years as reference). This suggests that prolonged duration of stay may be associated with a progressive deterioration in mental health among this population, possibly due to accumulated feelings of stress, hopelessness, and failure, in addition to challenges related to adaptation [3]. Similarly, Dunn et al. found significant differences in reported health problems based on length of residence, with long-term immigrants of 10 years or more being more likely to report at least one health issue [30].

The results show that migrants residing in the old town and in precarious habitats have significantly lower MCS scores compared to those living in the new town. On the other hand, those living in precarious and rural areas have higher PCS scores. 

The presence of significant differences in MCS scores between migrants living in the old town and those residing in the new town highlights the crucial importance of the housing environment on perceived QOL. Residents of the old town, often characterized by older and sometimes precarious housing, appear to experience lower levels of mental well-being. This could be attributed to various environmental factors such as population density, noise, pollution, and limited access to modern facilities and green spaces [4].

On the other hand, migrants living in precarious and rural areas present higher PCS scores. This observation could be explained by a less sedentary lifestyle, greater accessibility to outdoor spaces, and proximity to nature, which are often associated with better perceived physical health.

These results underscore the crucial role of housing conditions in the QOL of migrants, influencing both their psychological and physical well-being.

Finally, irregular Sub-Saharan migrants mention experiencing assaults, which is significantly associated with a deterioration in their QOL. Moreover, they exhibit significantly lower physical and mental scores compared to those who have not experienced violence, highlighting the profound impact of victimization experiences on QOL. The results of the present study showed that sexual violence (N=6) was observed more frequently among women than among men (four women reported a rape compared to two men). Similarly, the study by Leyva-Flores et al. [26] found that, among migrants passing through Mexico to the United States between 2009 and 2015, 21.6% of women reported rape compared to only 1.5% of men (N=3,535). Experiences of sexual violence tend to occur along certain transit routes where there is a significant presence of delinquent groups. In the case of women, some perceive unwanted sexual relations as one of the necessary tools to facilitate their passage. The violence is so frequent and widespread that migrants have come to accept it as part of the price to pay for migration [26].

Differences in PCS based on gender could also be explained by social perspectives and longevity. It has been shown that women live longer than men but experience more health problems [6]. In our study, 63% (N=191) of women compared to 47% (N=335) of men reported having had a health problem in the six months prior to the data collection date.

Moreover, women are more frequently exposed to stressful factors and conflicts related to their social roles. Women assume more responsibilities due to their roles as mothers and wives, and these responsibilities are often taken on at the expense of their own health [23].

Education level also influences health status. The proportion of illiteracy in our sample is 12% (N=191) among women compared to 9.6% (N=335) among men.

In summary, this analysis demonstrates how sociodemographic characteristics such as age, gender, length of stay, housing conditions, and lived experiences such as aggressions significantly influence the perceived QOL among irregular Sub-Saharan migrants in Morocco. These findings underscore the importance of specific interventions aimed at improving living conditions and providing tailored psychosocial support to this vulnerable population, thereby promoting their physical and mental well-being.

Our study is not without limitations. First of all, our sample was selected using non-probabilistic methods, which makes it susceptible to selection bias. It is not homogeneous in terms of gender distribution and continent of origin. However, it is not feasible to sample a population that is not subject to census and is difficult to identify. Moreover, the opportunity to interview 526 individuals from an irregular population is a rare privilege due to the challenges in recruiting individuals who are rebels and frustrated because of their situation.

Finally, this study has limitations related to its cross-sectional design, which does not reflect temporal changes in HRQOL unless repeated at another time. Despite these limitations, we believe that this study provides valuable information and analysis that contribute to understanding the situation of undocumented migrants living in conditions of significant social vulnerability.

## Conclusions

Sub-Saharan irregular migrants living in northern Morocco experience a low level of HRQOL. The study highlighted the influence of certain sociodemographic characteristics and life experiences on the physical and mental states of the migrants studied. The detailed analysis of different components of the SF-36 reveals diversity in their health perception, with significant implications for designing policies and social interventions aimed at improving their living conditions, security, and overall well-being and ensuring better recognition of their fundamental rights in host societies to facilitate their social integration.

Therefore, policymakers and stakeholders must engage in substantive efforts to prioritize interventions aimed at improving the determinants influencing migrant health.
